# Bacteria induce an amoeboid phase in coccolithophores that persists after bloom collapse

**DOI:** 10.1126/sciadv.adw7280

**Published:** 2025-08-27

**Authors:** Sophie T. Zweifel, Richard J. Henshaw, Oliver Müller, Johannes M. Keegstra, Samuel G. V. Charlton, Roberto Pioli, Clara Martínez-Pérez, Uria Alcolombri, Estelle Clerc, Roman Stocker

**Affiliations:** ^1^ Institute of Environmental Engineering, Department of Civil, Environmental and Geomatic Engineering, ETH Zürich, Zürich, Switzerland.; ^2^ Limnological Research Station, Department of Hydrology, University of Bayreuth, Bayreuth, Germany.; ^3^ A. Silberman Institute of Life Sciences, Faculty of Sciences, The Hebrew University of Jerusalem, Jerusalem, Israel.

## Abstract

Coccolithophores, including bloom-forming species, *Gephyrocapsa huxleyi* (formerly *Emiliania huxleyi*), contribute ~1 to 10% of phytoplankton biomass and are critical for oceanic biogeochemical cycles. *G. huxleyi* is a model system for investigating algal-bacterial-viral interactions and responses to environmental changes and follows a biphasic lifecycle with motile haploid and nonmotile diploid phases. Here, we report a third, “amoeboid” phase: Light and electron microscopy revealed haploid cells rapidly transitioning to an elongated amoeboid cell with reduced motility. Metamorphosis was triggered by exposure to bacteria isolated from *G. huxleyi* mesocosm blooms, but not by classical phytoplankton stressors including viral infection. The amoeboid phase persisted beyond the collapse of the haploid population and was only observed in the bloom-forming coccolithophore species *G. huxleyi* and *Gephyrocapsa oceanica* under conditions reminiscent of late-stage algal blooms. These findings highlight a previously uncharacterized life phase in this ubiquitous phytoplankton and suggest a bacteria-resilient morphotype following algal bloom collapse.

## Teaser

Metamorphosis in major phytoplankton species induced by exposure to associated bacteria.

## INTRODUCTION

Phytoplankton are responsible for about 50% of global primary production (*1*, *2*) and are, thus, a crucial component of ecosystem-scale biogeochemical processes. Prominent among the phytoplankton community are coccolithophores, a group of calcifying microalgae that account for nearly 10% of phytoplankton biomass (*3*, *4*) and are characterized by an intricate calcium carbonate shell comprising overlapping coccoliths. These autotrophs are vital components of both the organic and inorganic carbon pumps, sequestering over a billion tons of CaCO_3_ annually in the deep ocean upon cell death (*5*, *6*) and composing key links in biogeochemical cycling and ocean alkalinity dynamics (*7*). Given their global prominence, coccolithophores have been established as a model system to study a diverse range of marine processes, from physiological responses to ocean acidification in modern and historic oceans (*8*–*10*) to microscale processes including algal interactions with pathogenic bacteria and viruses, which have been linked to the termination of coccolithophore blooms through release of algicidal compounds and by triggering programmed cell death (*11*–*14*).

In contrast to some other phytoplankton such as diatoms (*15*), coccolithophores follow a biphasic lifecycle alternating between a diploid morphotype that is nonmotile and extensively covered in intricate heterococcoliths and a vegetatively growing haploid morphotype that is biflagellated, motile, and generally covered in simpler holococcoliths (*16*–*21*). *Gephyrocapsa huxleyi* [formerly known as *Emiliania huxleyi*; (*22*)] is a notable exception with its haploid stage lacking calcification entirely (*23*). The two primary bloom-forming coccolithophores, *G. huxleyi* and *Gephyrocapsa oceanica*, are ubiquitous in the euphotic zone in almost all ocean regions (*24*, *25*), often forming kilometer-scale blooms that are visible from space (*24*, *26*–*28*). These two species alone account for more than a third of oceanic calcium carbonate formation (*29*). A critical component to shortening *G. huxleyi* blooms or causing their collapse is a rise in large virus-like particles, now known as *G. huxleyi* viruses (EhVs) (*30*–*33*), which can trigger a shift in metamorphosis of a subpopulation of the virally susceptible diploid into a virally resistant biflagellated noncalcifying diploid morphotype, closely resembling the morphology of haploid cells (*34*–*36*). In the case of *G. huxleyi*, earlier studies have occasionally noted the presence of a third life phase (*16*, *17*, *37*). This “amoeboid” morphotype was first reported as a fusiform-shaped cell with “wriggling” motility (*37*). A later study found these amoeboid cells within stationary diploid *G. huxleyi* cultures and proposed that they had lost their cell wall (*16*). Beyond these reports, this elusive morphotype has remained unexplored, in particular as to its triggers and ecological function.

Here, we report that amoeboid metamorphosis was only induced when certain *G. huxleyi*–associated bacteria were added at high densities to both haploid bloom-forming coccolithophores *G. huxleyi* and *G. oceanica* cultures in stationary phase. After bacterial exposure, *G. huxleyi* cells from late-stage cultures transform within minutes into a motile, noncalcified, amoeboid with apparent resilience toward bacteria. We extensively characterize the amoeboid phase of *G. huxleyi* using a combination of flow cytometry and time-resolved video, electron, and phase modulation microscopy. We identified that high bacterial concentrations and stationary phase bloom-forming algal cells are required for triggering the metamorphosis into the amoeboid phase, finding them to be compatible with localized conditions present within the demise of algal blooms. The amoeboid phase may thus represent an important life cycle stage in the bloom dynamics of coccolithophores, furthering our understanding of the complex processes underpinning algal blooms.

## RESULTS

### Haploid bloom-forming coccolithophores can undergo a rapid morphological transition to a motile amoeboid phase

**Fig. 1.**
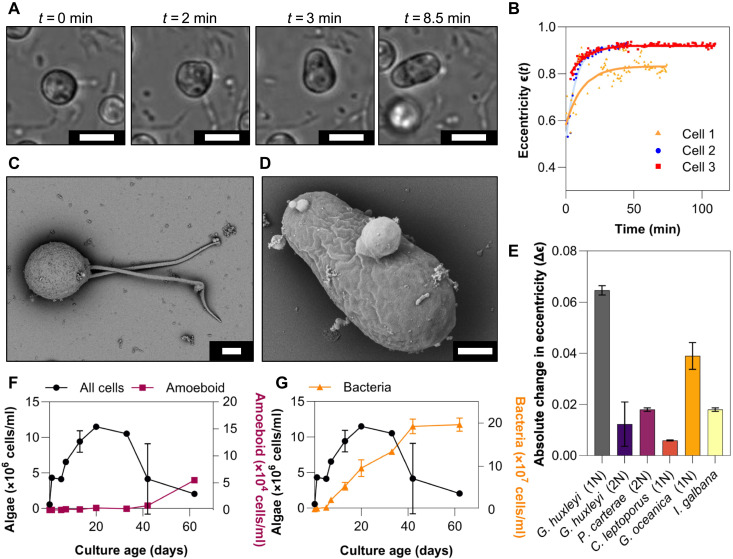
Metamorphosis haploid to amoeboid phase in coccolithophores. (**A**) Light microscopy time-lapse images of a haploid *G. huxleyi* cell in the transition to an ellipsoidal amoeboid cell (scale bars, 5 μm). (**B**) Change in eccentricity (perfect sphere eccentricity ϵ=0 ) over time for three cells (colored points) cell morphology transitions from broadly spherical to ellipsoidal with a characteristic timescale of 15 min [extracted from the exponential fits (lines)]. (**C**) Scanning electron microscopy (SEM) image of a *G. huxleyi* haploid biflagellated cell (scale bar, 1 μm). (**D**) SEM image of a *G. huxleyi* amoeboid cell (scale bar, 1 μm). (**E**) Change in eccentricity after exposure to the bacteria, *Marinomonas* 5a1, and before exposure Δϵ=∣ϵ(treatment)−ϵ(control)∣ for various haptophyte strains, with data plotted as the mean with SE bars and *n* = 3 biological replicates. (**F**) Cell counts as a function of time for an untreated xenic haploid *G. huxleyi* culture for all algal cells (black circles, left axis) and amoeboid cells (pink squares, right axis), with data plotted as the mean with SE error bars and *n* = 3 biological replicates. (**G**) Cell counts as a function of time for xenic haploid *G. huxleyi* culture for all algal cells (black circles, left axis) and total bacterial cells (orange triangles, right axis), with data plotted as the mean with SE error bars and *n* = 3 biological replicates.

Using time-lapse microscopy, we observed a previously uncharacterized morphotype among xenic haploid cultures of the model coccolithophore *G. huxleyi* (RCC1217), whereby the cell rapidly elongates from the typical spherical shape to an ellipsoidal form that we hereafter refer to as the amoeboid phase (Fig. 1A and movie S1). This phase has previously been briefly reported (*16*, *37*, *38*), where elongated cells were observed to undergo a wriggling motion (*37*). Electron microscopy imaging later captured an image of an elongated cell body (*16*), yet neither a detailed characterization nor the origin of this phase was pursued. Using single-cell tracking and image analysis (Fig. 1B), we found that the transition occurs rapidly, with the cell’s eccentricity markedly increasing over a 15-min period before stabilizing. The cell ceases motility during the transformation and resumes it thereafter (movie S2). Scanning electron microscopy (SEM; Materials and Methods) images of the haploid (Fig. 1C) and amoeboid (Fig. 1D) cells confirm the stark contrast in morphology between the two phases. Using energy-dispersive x-ray (EDX) spectroscopy (Materials and Methods), we discovered that the amoeboid phase lacks calcification, evidenced by the absence of characteristic calcium carbonate EDX peaks (fig. S1). Measurements of the zeta potential showed no significant differences in cell surface charge between haploid and amoeboid cells (Materials and Methods and fig. S2A); however, microbial adhesion to hydrocarbons (MATH) assay revealed a 39 ± 2% increase in surface hydrophobicity in the amoeboid phase (Materials and Methods and fig. S2B), which can alter cell recognition as well as enhance stickiness and cell aggregation (*39*, *40*).

We observed considerable amoeboid cell formation only in the haploid morphotypes of the two bloom-forming coccolithophores *G. huxleyi* and *G. oceanica*, and not for example in diploid *G. huxleyi* or in the closely related haptophyte *Isochrysis galbana* (Fig. 1E and fig. S3). While small changes in eccentricity were detected in these other strains, microscopy revealed no cells resembling the amoeboid morphotype, suggesting that the observed eccentricity shifts likely result from minor morphological variation and/or segmentation limitations in the cell tracking algorithm. Although we did not observe amoeboid formation in diploid *G. huxleyi* under the conditions or with the bacterial strains tested in this study, it remains possible that other bacterial partners, environmental stressors, and/or specific physiological states may trigger a similar transition.

Monitoring *G. huxleyi* cultures via flow cytometry revealed that the amoeboid cells could be distinguished from the normal haploid population by a shift in forward scatter (FSC) area (fig. S4). SYBR-Green DNA staining and flow cytometry showed that relative cellular DNA content of haploid and amoeboid populations is similar, suggesting that the transition from the haploid phase to the amoeboid phase conserved the ploidy of the original haploid phase (fig. S4). The amoeboid phase becomes increasingly prominent (up to 3% of the original population) as cultures age into stationary and declining phase (Fig. 1F), consistent with previous observations of amoeboid cells being present in older cultures (*16*). Furthermore, flow cytometry measurements of the separate populations of xenic haploid *G. huxleyi* show an increase in absolute amoeboid population, coinciding with a peak in bacterial cell number and increasing further as the overall algal culture collapses. Specifically, the *G. huxleyi* culture density declines from 1.1 × 10^7^ cells/ml on day 20 to 2 × 10^6^ cells/ml by day 62 (Fig. 1G). This reduction (9 × 10^6^ cells/ml) in overall cell numbers cannot be accounted for by the relatively modest rise in amoeboid cell numbers (~5 × 10^4^ cells/ml), pointing at cell death as the most likely explanation for the rapid decline of the haploid population.

**Fig. 2.**
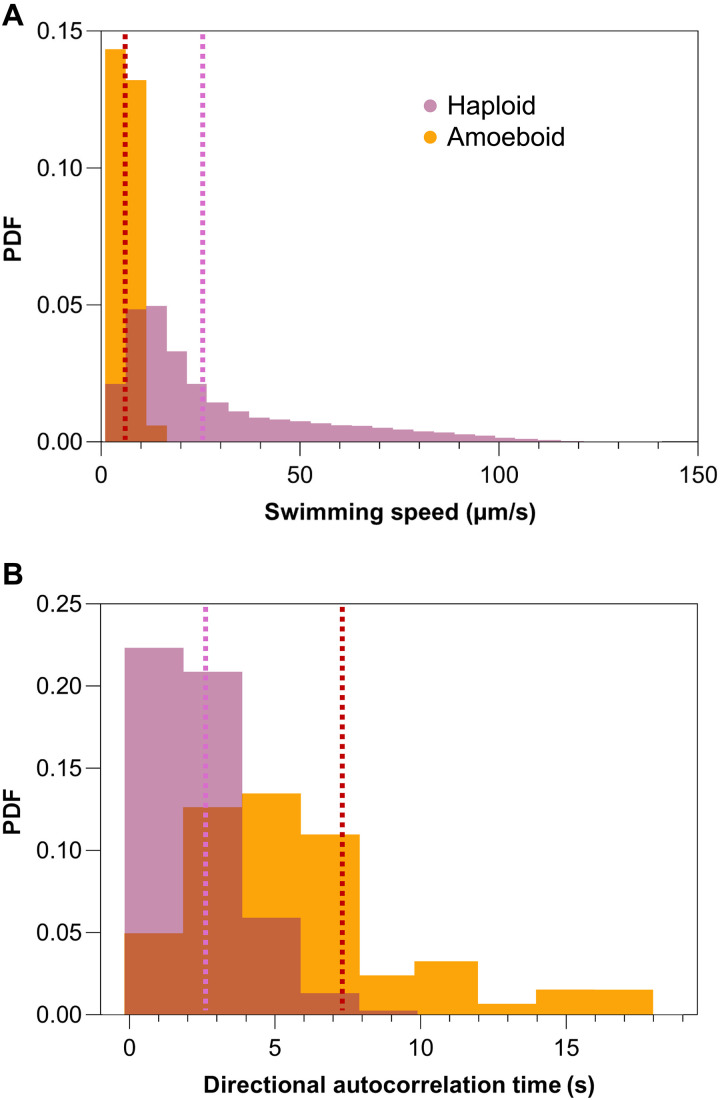
Motility of *G. huxleyi* haploid and amoeboid cells. (**A**) Probability density functions (PDF) of swimming speed of *G. huxleyi* haploid cells (purple) and amoeboid cells (yellow) with speeds of 26.3 ± 1.8 µm/s and 7.1 ± 0.1 μm/s (means ± SEM), respectively, with *n* = 8 biological replicates. (**B**) Velocity autocorrelation of the haploid and amoeboid phases, with an autocorrelation time of 2.8 ± 0.1 s and 7.2 ± 0.9 s (means ± SEM) for the haploid and amoeboid respectively, with *n* = 8 biological replicates. Average speeds and autocorrelation times indicated with dotted purple (haploid) and dotted orange (amoeboid) lines.

Metamorphosis into the amoeboid phase was accompanied by a considerable change in motility. Haploid cells swim in a meandering, looping trajectory with a rapidly decorrelating swimming direction, reflected in an average autocorrelation time of 2.8 ± 0.1 s and a mean speed of 26.3 ± 1.8 μm/s (Fig. 2). In contrast, amoeboid cells have an average autocorrelation time of 7.2 ± 0.9 s, thus changing direction approximately three times less often than haploid cells per unit time (Fig. 2B and movie S2), with an average speed of 7.1 ± 0.1 μm/s (Fig. 2A), approximately a quarter of the average haploid swimming speed. Furthermore, the swimming speed distribution is narrower for the amoeboid cells in comparison to haploid cells, with SDs of 0.3 and 5.6, respectively. Although we could not detect the presence of flagella with either light or electron microscopy, during motion, the cell body can be clearly seen to rotate (movie S2) in a manner similar to other flagellated cells, which combined with the asymmetrical body shape gives the appearance of wriggling, as noted in previous records of amoeboid cells (*37*). However, it remains unclear whether and how the flagella beat patterns are altered by this metamorphosis.

### Amoeboid cells maintain photophysiology and are not induced by typical environmental triggers

**Fig. 3.**
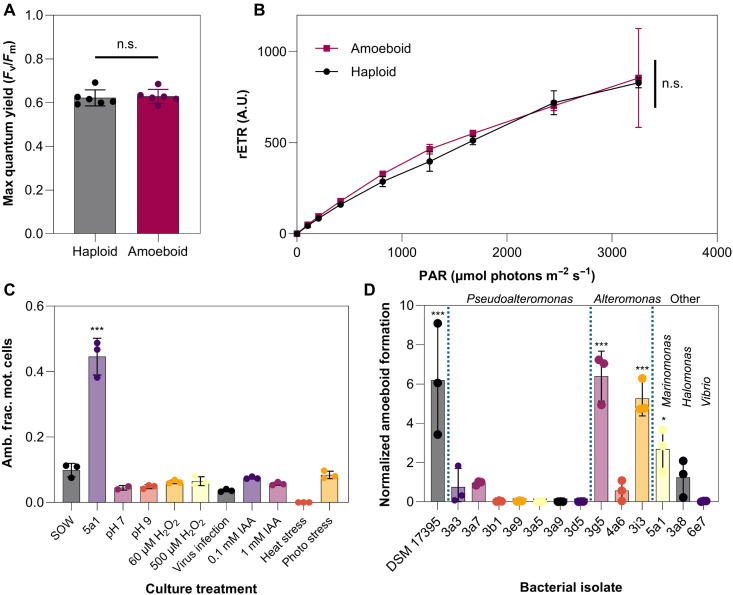
Amoeboid cells maintain photophysiology and metamorphosis is only triggered by exposure to environmentally associated bacteria and not other environmental stressors. PAM measurements of haploid (gray) and amoeboid cells (purple, induced via exposure to bacterial strain *Marinomonas* 5a1); neither the (**A**) maximum quantum yield nor (**B**) relative electron transport rate (rETR) differs between the amoeboid and haploid phases. Data plotted as the mean with SE error bars with *n* = 6 biological replicates. A two-tailed *t* test showed no statistically significant differences (*P* < 0.05) between the two phases for both the maximum and effective quantum yields (across all PAR values). n.s., not significant; A.U., arbitrary unit. (**C**) The amoeboid fraction among motile cells (Amb. frac. mot. cells) due to exposure of *G. huxleyi* haploid cells to different environmental stressors. Only exposure to bacteria (here, the isolate *Marinomonas* 5a1) induced the formation of a significantly greater number of amoeboid cells in comparison with the synthetic ocean water (SOW) control (no stressors applied). Data shown as the mean with SE error bars and *n* = 3 biological replicates. A one-way analysis of variance (ANOVA; *P* < 0.05, *F* = 111.4) was carried out followed by a one-tailed post hoc Tukey test applied for each treatment against the SOW (*P* < 0.05). (**D**) Relative amoeboid population, after exposure of haploid *G. huxleyi* cells to environmentally associated bacterial isolates (*45*) from five bacterial genera: *Pseudoalteromonas*, *Alteromonas*, *Marinomonas*, *Halomonas*, and *Vibrio*, and exposure to the *G. huxleyi* pathogen *P. inhibens* (DSM 17395). Amoeboid induction is shown to be bacterial-strain dependent, with significant induction in response to 4 of the 14 strains. Data are normalized against the SOW treatment and plotted as the mean with SE error bars and *n* = 3 biological replicates. A one-way ANOVA (*P* < 0.05, *F* = 19.67) was carried out followed by a one-tailed post hoc Tukey test applied for each treatment against the SOW (*P* < 0.05).

To assess the cell fitness of amoeboid cells, quantum yields were measured using pulse amplitude modulation (PAM) microscopy. Measurements of the maximum quantum yield and relative electron transport rate (rETR) are commonly used as proxies for cell health and photosynthetic activity, respectively (*41*, *42*). A comparison between haploid and amoeboid cells revealed no significant difference in either the maximum quantum yield (Fig. 3A) or rETR (Fig. 3B), indicating that cells maintain comparable photosynthetic fitness after transformation to the amoeboid phase (*43*, *44*). We note that the amoeboid cultures used for these measurements were not composed entirely of amoeboid cells; however, analysis of the full distribution of quantum yield values within the population (fig. S5) did not reveal any distinct subpopulations, suggesting that any remaining haploid cells in the culture had similar photosynthetic performance. Furthermore, while tracking of individual cells through transition from haploid to amoeboid form was not possible, the lack of a bimodal distribution in the quantum yield data indicates that there is no major fitness difference between transitioning haploid cells and their amoeboid counterparts under the conditions tested.

We performed a systematic screening of common environmental stressors to determine the trigger of the amoeboid transformation. Xenic haploid cultures were exposed to different potential stressors (Materials and Methods), including rapid changes in pH, temperature increases, viral infection, oxidative stress, photoinduced stress, and indole-3-acetic acid [IAA; at either growth-enhancing (0.1 mM) or growth-inhibiting (1 mM) concentrations] (*11*). Cultures were then maintained in pre-exposure growth conditions and monitored over 24 hours (Fig. 3C). None of these stressors triggered a significant amoeboid response, indicating that the amoeboid phase transition is not a generalized stress response. Instead, a significant fraction of the population underwent metamorphosis into the amoeboid phase when a haploid culture was inoculated with high densities of an environmentally relevant bacterial strain, *Marinomonas* 5a1, previously isolated from a *G. huxleyi* bloom in a mesocosm experiment (*45*) in Bergen (Norway) (Fig. 3C). This observation is consistent with our finding that amoeboid cells are induced within stationary phase xenic haploid cultures with a high density of bacteria (Fig. 1, F and G). The metamorphosis into the amoeboid phase also does not appear to be related to a change in predatory strategy: While the haploid cell contains acidic compartments for bacterial phagocytosis (*46*), staining of both the diploid and amoeboid demonstrated the absence of such compartments (fig. S6 and Materials and Methods).

### Amoeboid formation is triggered by bacterial exposure resembling bloom collapse conditions

To examine the specificity of the impact of bacteria on the transformation from haploid to amoeboid phase, haploid *G. huxleyi* cultures were independently inoculated with 13 bacterial strains isolated from the same mesocosm experiment in Bergen (*45*). Amoeboid cells were characterized through light microscopy and particle tracking (Materials and Methods) and identified through their differences in motility and morphology (Figs. 1, C and D, and 2, A and B). Aside from the above identified *Marinomonas* 5a1 strain (Fig. 3C), two *Alteromonas* strains (3g5 and 3i3) also triggered considerable amoeboid induction in *G. huxleyi* (Fig. 3D). No response was observed to any of the seven isolated *Pseudoalteromonas* strains. Significant amoeboid induction was further observed with the known *G. huxleyi* pathogen *Phaeobacter inhibens* (DSM 17395, found in the microbiome of *G. huxleyi* isolated from the Galician coast) (*11*, *38*, *47*, *48*). These observations demonstrate a specificity in the metamorphosis trigger, with only particular coccolithophore-associated bacterial strains inducing amoeboid formation.

**Fig. 4.**
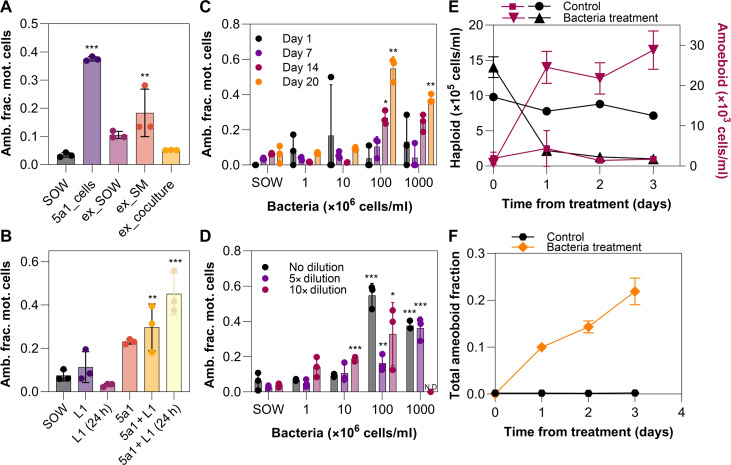
Algal and bacterial conditions required for bacterial-induced amoeboid formation in *G. huxleyi*. (**A**) Fraction of amoeboid cells among motile cells (Amb. frac. mot. cells) when haploid *G. huxleyi* cultures were exposed to exudates from *Marinomonas* 5a1 under three treatments: SOW, spent medium of *G. huxleyi*, and *G. huxleyi* culture. (**B**) Amb. frac. mot. cells under nutrient conditions: control (SOW) versus addition of inorganic L1 nutrients (phosphorus, nitrate, silicate, trace metals, and vitamins) added either immediately before or 24 hours before incubation with 5a1. (A and B) Data are means ± SE (*n* = 3 biological replicates). One-way ANOVA [*P* < 0.05, *F* = 38.97 for (A) and 16.85 for (B)] followed by one-tailed post hoc Tukey test versus SOW (*P* < 0.05). (**C**) Amb. frac. mot. cells at four *G. huxleyi* culture time points: day 1 (black, pre-exponential), day 7 (purple, exponential), day 14 (pink, stationary), and day 20 (orange, declining), with different supplemented bacterial concentrations. Significant induction only occurred in stationary/older cultures with >10^8^ cells/ml bacteria. (**D**) Amb. frac. mot. cells under varying algal:bacterial ratios (starting *G. huxleyi* concentration, 10^6^ cells/ml). Significant induction occurred at high bacterial concentrations and at lower ones when algal culture was diluted. (C and D) Data shown as means ± SE (*n* = 3). Two-way ANOVA (*P* < 0.05), followed by one-tailed post hoc Tukey test versus SOW (*P* < 0.05). (**E** and **F**) Flow cytometry counts (E) of haploid and amoeboid *G. huxleyi* cells in control (black circles, pink squares) versus cultures with 5a1 (black triangles, pink inverted triangles). (F) Resulting amoeboid cell fraction in control (black circles) and 5a1-inoculated cultures (orange diamonds). Data are means ± SE (*n* = 3).

To investigate the nature of the algal-bacteria interaction responsible for triggering the transformation, *Marinomonas* 5a1 was used to prepare three treatments. Overnight cultures of 5a1 were washed and incubated in (i) unsupplemented synthetic ocean water (SOW) medium, (ii) a stationary phase haploid *G. huxleyi* culture, and (iii) filtered medium from a stationary phase haploid culture, respectively. After 24 hours of incubation, the three cultures were filtered to remove all cells, and the filtrates were then supplied to three stationary-phase haploid cultures of *G. huxleyi*. The first and third treatments triggered amoeboid formation (Fig. 4A), demonstrating that the presence of bacterial exudates alone is sufficient to induce transformation, without the need for physical contact between the bacteria and algae. In contrast, filtrates from the second treatment did not induce any significant amoeboid formation, suggesting that potential chemical triggers were either degraded or consumed by the algae during the incubation stage.

Further experiments indicate that the transformation is not a starvation response. Inoculating stationary phase haploid cultures with both *Marinomonas* 5a1 and a nutrient supplement (containing organic nutrients, phosphate, nitrate, silicate, trace metals, and vitamins, so as to supplement the medium back to starting concentrations of the L1 algae growth medium; Materials and Methods) still resulted in amoeboid formation (Fig. 4B). This indicates that metamorphosis is not induced by the consumption of the initially supplied inorganic nutrients by either bacteria or algae.

Induction of the amoeboid phase primarily occurs in conditions typical of those following a bloom collapse. Amoeboids were originally significantly detected with high densities of both algae and bacteria (10^6^ and 10^9^ cells/ml, respectively). To test for a dependence of amoeboid metamorphosis on algal cell age, four different ages (1, 7, 14, and 20 days old) of haploid *G. huxleyi* cultures were inoculated with bacterial cultures of different concentrations, from 10^6^ to 10^9^ cells/ml (Fig. 4C). Significant amoeboid induction was observed in cultures older than 14 days (i.e., stationary and older; fig. S7) in the two highest starting bacterial concentrations (10^8^ and 10^9^ cells/ml). In a further experiment, a 20-day-old haploid culture with starting density of 10^6^ cells/ml was divided into triplicate cultures and diluted by either 10×, 5×, or no dilution and then inoculated with different bacterial concentrations (Fig. 4D). Amoeboid formation in these diluted *G. huxleyi* cultures was observed for initial bacterial concentrations of 10^6^ cells/ml and higher. Together, these results identify two important aspects of the metamorphosis. First, amoeboid formation is not induced in young cultures regardless of starting bacterial densities. Second, the formation of amoeboid cells at algal concentrations of 10^5^ cells/ml and bacterial concentrations of 10^6^ cell/ml, matching concentrations reported in naturally occurring *G. huxleyi* blooms (*24*, *49*), indicates that this third life phase may be ecologically relevant in the ocean in late-stage algal blooms.

Tracking both the amoeboid and haploid phases in *G. huxleyi* over time with flow cytometry (Materials and Methods) showed that exposure to *Marinomonas* 5a1 resulted in a small subpopulation, consisting of ~3% of the original haploid population, transitioning to amoeboid cells within 3 days (Fig. 4E). This subpopulation persisted under the inducing bacteria-rich conditions. In contrast, cell counts of the initial haploid population simultaneously decreased by 93% within 3 days (Fig. 4E), resulting in 21% of the final algal population being represented by amoeboid cells (Fig. 4F).

## DISCUSSION

In this study, we have characterized an additional life phase in bloom-forming coccolithophores. While this life phase had been briefly reported previously (*16*, *38*), both a detailed description and identification of the triggering mechanism had been lacking. Our experiments show that, upon exposure to particular bacterial strains, stationary-phase *G. huxleyi* haploid cells undergo a rapid morphological transformation over a timescale of 15 min, during which they elongate from nearly spherical to a 3:1 ellipsoid (Fig. 1, A and B) and reduce their average swimming speed by 73% (Fig. 2A). PAM measurements demonstrate that amoeboid cells retain the same photophysiology as the haploid cells (Fig. 3, A and B), indicating that amoeboids maintain comparable photosynthetic fitness to the original haploids. We find that a suite of environmental stressors, including inorganic nutrient starvation (Fig. 4B), viral infection, oxidative stress, pH changes, and photostress, all fail to induce this metamorphosis (Figs. 3C and 4B), while exposure to several species of environmentally relevant bacteria do. This transformation does not require physical contact between algae and bacteria (Fig. 4A). During exposure to high bacterial concentrations which trigger this metamorphosis, the amoeboid population persists after the crash of the haploid population, reaching up to 21% of the total cell numbers (Fig. 4, D to F). Because we show that the *Marinomonas* isolate has algicidal effects on coccolithophores, as do some of the other bacterial species tested (*11*, *47*, *48*), we hypothesize that the persistent morphotype may confer resilience against pathogenic bacteria under bloom-collapse conditions.

The environmental conditions that trigger the transition from haploid to amoeboid phase are commensurate with those of the harsh conditions seen at the end of algal blooms (*24*, *50*–*53*). First, induction was only observed in bloom-forming coccolithophore species (*G. huxleyi* and *G. oceanica*) and not in other common coccolithophores, including the closely related but non–bloom-forming haptophyte *I. galbana* (Fig. 1E). Second, this metamorphosis occurs predominantly in older stationary and declining haploid cultures (Figs. 1, F and G, and 4C) in the presence of high densities of certain bacterial strains, including a well-known *G. huxleyi* pathogen (*P. inhibens*) and strains isolated from a mesocosm in which a coccolithophore bloom had been induced (Fig. 2D) (*45*). This previously reported association with stationary phase (*16*) is further supported by results from untreated xenic haploid cultures, where the rise in amoeboid cell numbers coincides with the onset of stationary phase, increase in bacterial abundance, and rapid decline of the haploid population (Fig. 1, F and G). Amoeboid induction occurring only with stationary phase cultures suggests that the physiology of the algal cell is a pivotal factor in enabling the transformation into the amoeboid phase. Future studies should aim to address the physiological requirements and metabolic costs of this rapid transition, which would likely provide direct insight into the internal processes driving this response. One potential explanation for the persistence of amoeboid cells in bacteria-rich conditions could be related to the changes in surface hydrophobicity associated with this metamorphosis (fig. S2), which could impair bacterial recognition of amoeboid cells (*54*–*56*). Another possible explanation is the exudate composition of amoeboid cells could be shifted compared to haploid exudates, thus avoiding detection by bacteria, something that would require a dedicated metabolomic investigation.

A primary distinction between the haploid and the amoeboid phases is the observed persistence through exposure to conditions generated by bacteria (Figs. 1, F and G, and 4E). Upon exposure to *Marinomonas* 5a1, haploid cell numbers decline by 93%, from 1.4 × 10^6^ to 1 × 10^5^ cells/ml. In parallel, amoeboid cell numbers exhibit a 35-fold increase, rising from 7 × 10^2^ to over 2.5 × 10^4^ cells/ml, and remain at this elevated abundance throughout the 3-day observation period (Fig. 4E). In an ecological context, our results indicate that amoeboid metamorphosis could be a potential survival response to the harsh bacteria-rich conditions locally present during the culmination of algal boom collapse. It is worth noting that we show that the virally resistant haploid cells (*34*) remain susceptible to bacteria-generated harsh conditions (Fig. 4E), underscoring a key ecological challenge for *G. huxleyi*: While the haploid phase offers protection against viral lysis, it does not confer resistance to the increasingly bacterially dominated environment following bloom collapse. Therefore, to persist beyond this phase of ecological succession, *G. huxleyi* must deploy an additional survival strategy, such as, we suggest, entering the amoeboid state.

While we observe amoeboid formation at cell concentrations relevant to those found in naturally occurring *G. huxleyi* blooms (*24*, *49*) (10^5^ cells/ml for algae and 10^6^ cells/ml for bacteria), these reported environmental cell densities are for total *G. huxleyi* populations and not just haploid cells, as we have tested here. Although haploid-specific counts are lacking, noncalcifying *G. huxleyi* cells have been detected at concentrations of 1 × 10^3^ cells/ml in natural blooms, comprising a mixture of haploids and noncalcifying diploids (*23*). We propose that, while these interactions and transformations may be rare at the bulk scale, they could play a relevant ecological role within localized environments across the heterogeneous conditions of a bloom event.

Here, we have identified bacterial conditions as a trigger for the amoeboid phase, although this finding does not exclude the existence of other environmental triggers for the amoeboid phase. Previous reports have described amoeboid-like cells in stationary *G. huxleyi* diploid cultures (*16*), which were not observed here, indicating alternative triggers or conditions for amoeboid formation or, potentially, also reflecting the presence of previously documented small haploid subpopulations within diploid cultures that may have undergone the transition as we have described. Similarly, while *G. oceanica* haploids displayed capacity for amoeboid formation, further work would be needed to determine if the diploid phase of this species could also undergo a comparable transformation under different conditions. These findings highlight the potential for species- and ploidy-specific responses and underscore the importance of future studies to explore a broader range of bacterial interactions and environmental cues.

**Fig. 5.**
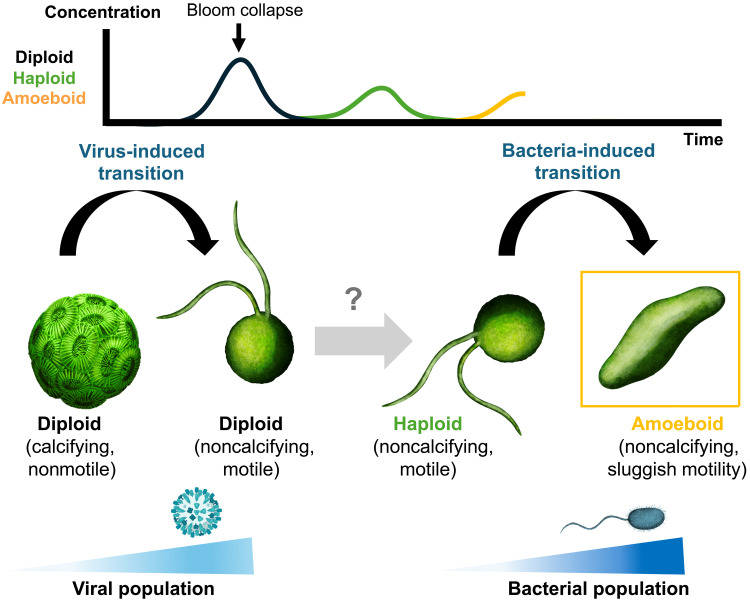
Proposed morphological cascade of *G. huxleyi* during algal bloom collapse in response to sequential environmental stressors. During bloom development, the diploid population reaches peak abundance, followed by a sharp rise in virus-like particles that lyse diploid cells. This viral infection has been shown to induce a biflagellated, noncalcifying diploid morphotype that is resistant to viral lysis (*23*, *33*, *34*, *36*). A subsequent transition from this diploid flagellated form to the haploid phase has been hypothesized (*23*, *34*, *36*), but not yet demonstrated, and is indicated here with a grayed-out arrow and question mark. We provide experimental evidence for the final step of this cascade, showing that haploid cells can rapidly transition into an amoeboid, bacterially resistant morphotype in response to bacterial stress during late bloom collapse. Note that the relative magnitudes of population peaks are illustrative and not to scale.

Regardless of the root cause, the transition into a third life phase expands the underlying mechanisms driving algal blooms and their collapse, particularly as the transition into the amoeboid phase is not the first instance of coccolithophores responding to rapidly changing environmental conditions. The general progression of bloom dynamics in coccolithophores has been well-documented (*24*, *49*, *57*): It starts with an increase in the diploid population and is followed by a rise in viral particles that triggers a formation of a virally resistant subpopulation of flagellated noncalcifying cells (*34*–*36*). The virally lysed susceptible diploid cells then provide organic matter to support a subsequent bacterial bloom (*58*, *59*). It is well established that the haploid phase of *G. huxleyi* is resistant to viral lysis (*13*, *14*, *30*, *33*, *36*); however, a direct transition from the diploid to the haploid phase explicitly triggered by viral infection, while hypothesized (*36*), has not yet been demonstrated. Instead, it has been shown that viral exposure induces the formation of a biflagellated, noncalcifying diploid morphotype, which closely resembles the haploid in appearance but crucially not its ploidy (*36*). This demonstrates two remarkable morphological transitions by *G. huxleyi* in distinct periods of a bloom event: transitioning to independent resistant forms when exposed to elevated concentrations of viruses and bacteria, respectively. If the missing connection between the virally resistant diploid and haploid morphotypes is observed, then this would complete a complex morphological cascade that spans the duration of a bloom event (Fig. 5). Identifying the missing link between diploid and haploid transitions remains a key open question in the study of coccolithophores, especially now in the context of the amoeboid transition and this proposed morphological cascade.

A natural question prompted by our work is how the coccolithophore life cycle proceeds after the amoeboid phase. We isolated and observed individual amoeboid cells away from the bacterial population in microfluidic isolation devices with medium replacement; however, we did not observe a reversal of the metamorphosis or cell growth under these conditions. While we have not observed a transition from the amoeboid to either the haploid or diploid phases, this would close the expanded life cycle of *G. huxleyi.* If such a transition exists, then it likely requires specific environmental triggers that have so far proved elusive under laboratory conditions. Further work will also be required to determine whether the transition to the amoeboid phase is directly triggered by the bacteria or is an active defense response against the bacteria, which signaling factors drive it, and what biomechanical mechanism underpins the metamorphosis.

We expect the amoeboid phase to be more prevalent than the lack of reports to date would imply. It would be intriguing and further cement the ecological relevance of amoeboids to search for signs of this life phase in existing environmental field data such as Imaging FlowCytobot data (*60*). The robustness of the transformation makes it appear likely that amoeboid cells have been overlooked because of the marked morphological differences compared to the traditionally studied diploid and haploid cells. Given the rise of algal bloom events linked with ongoing rising ocean temperatures (*61*, *62*), we anticipate that these results will promote future investigations into the interspecies interactions shaping algal bloom dynamics and, further, our understanding of these key microbial players on the global scale.

## MATERIALS AND METHODS

### Algal strains and growth conditions

All algae strains were purchased from the Roscoff Culture Collection (Roscoff, France), with the exception of CCMP3266 that was purchased from the National Center for Maine Algae and Microbiota Bigelow Laboratory (Maine, USA). All algae strains were grown in L1-supplemented SOW (pH 8) (*63*). Haploid and diploid *G. huxleyi* strains RCC1217 and CCMP3266 (also known as RCC1216), respectively, *G. oceanica* haploid strain RCC1315, *I. galbana* strain RCC1353, and *Pleurochrysis carterae* diploid strain RCC6317 were cultured at 18°C on a 14:10-hour diurnal cycle at a light intensity of 100 μmol m^−2^ s^−1^. *Calcidiscus leptoporus* haploid strain RCC1131 was cultured at 18°C on a 14:10-hour diurnal cycle at a light intensity of 60 μmol m^−2^ s^−1^. *Coccolithus braarudii* diploid strain RCC1200 was cultured at 14°C on a 12:12-hour diurnal cycle at a light intensity of 30 μmol m^−2^ s^−1^.

### Bacterial growth, amoeboid induction conditions, and exudate preparation

*P. inhibens* (DMS 17395) was purchased from the German Collection of Microorganisms and Cell Cultures (DSMZ, Braunschweig, Germany). All other bacterial strains were previously isolated from an induced mesocosm *G. huxleyi* bloom in Bergen (*45*) and preserved in 20% glycerol solution at −80°C. Bacteria were inoculated in Marine Broth medium (Difco, CAS 10043-52-4) from frozen stocks and grown overnight in a shaking incubator at 30°C with constant agitation (200 rpm). Cultures were collected at optical density (OD) of 1.70 to 1.80 and were washed and resuspended three times (4000 relative centrifugal force, for 5 min) with SOW. Algal cultures were then inoculated with the washed bacterial cultures at a cell ratio of 100:1 (unless otherwise stated) and incubated at the relevant temperature, diurnal light cycle, and light intensity for each of the algae strains tested (specified above, see the “Algal strains and growth conditions” section) for 24 hours to induce amoeboid formation.

Exudates were prepared by first growing the isolated strain 5a1 as described above and then inoculating three different treatments with a concentration of 10^9^ cells/ml (measured with flow cytometry, see the “Flow cytometry and staining” section): (i) fresh SOW medium, (ii) filtered (0.2-μm filter, Filtropur) spent medium from stationary *G. huxleyi* haploid culture, and (iii) a stationary *G. huxleyi* haploid culture. All treatments were incubated at 18°C on a 14:10-hour light:dark cycle at 100 μmol m^−2^ s^−1^ for 24 hours. After incubation, all bacterial and algal cells were removed via filtration (0.2-μm filter, Filtropur), and the resultant filtrate inoculated into replicate algal cultures (see the “Algal strains and growth conditions” section) with 1:10 *G. huxleyi* culture:exudate volume ratio (Fig. 4A).

### Preparation of algal stressors

*G. huxleyi* lytic virus EhV-201 (*64*) was provided by A. Vardi and maintained in exponential phase axenic diploid *G. huxleyi* (CCMP3266) cultures. *G. huxleyi* haploid cultures were infected with EhV-201 at multiplicity of infection of 1 (*33*, *65*). High- and low-pH conditions (pH values of 9 and 7, respectively) were created by the addition of NaOH and HCl to haploid cultures, respectively. *G. huxleyi* haploid cultures were thermally stressed by rapidly raising the culture temperature from 18° to 50°C for 10 min. Photostress was imposed by subjecting the cells to epi-illumination at 587-nm light supplied from a Sola Light Engine Lumencor at full power (~4 W) equipped to an inverted microscope [Nikon Ti-E; 10× 0.3 numerical aperture (NA) objective and mCherry filter cube]. Oxidative stress was induced by adding hydrogen peroxide (60 μM), and IAA (Merck, CAS 87-51-4) was prepared in 50% ethanol-water solutions and diluted to final concentrations of 0.1 and 1 mM IAA in the haploid *G. huxleyi* culture with a final ethanol concentration of 1% (*48*).

### Microscopy and single-cell tracking

Imaging chambers were prepared by separating one standard glass microscopy slide (Avantor VMR) and a standard cover glass slide (Number 1.5, Epredia) with a 1-mm-thick gasket fabricated by punching a polydimethylsiloxane (Dow Corning SYLGARD 184) sheet with an 8-mm-diameter biopsy punch. Samples were gently pipetted into the imaging chambers before sealing with the cover glass slide. Once loaded, imaging chambers were incubated at 18°C for 24 hours on a 14:10-hour light:dark cycle at 100 μmol m^−2^ s^−1^. Imaging of the samples was performed using phase-contrast microscopy (Nikon Ti-E; 10× 0.3 NA objective). Videos for cell tracking and shape analysis were recorded at 25 frames per second using a complementary metal-oxide semiconductor camera (Orca-Flash 4.0, Hamamatsu).

Cell tracking was conducted using TrackPy (v.0.6.1) (*66*), described previously for tracking bacterial movement (*45*). The background of each image was removed by computing the absolute difference of each pixel value with the median intensity value per pixel over the full length of the video (30 s, 600 frames). Particle detection was performed on the absolute intensity difference with a particle size of 17 pixels and a minimum distance of 51 pixels between particles. For the assembly of trajectories a maximum displacement of 17 pixels and maximum disappearance of two frames was allowed. Resulting cell velocities were calculated with a five-frame moving-average. Trajectories shorter than 10 frames were discarded. For the autocorrelation analysis, the cellular positions were processed with a second-order Savitzky-Golay filter with a time window of five frames, and the change in angle was computed from the filtered positional data. The autocorrelation function was then computed using the Python Numpy function correlate on the angle time series of each trajectory. The correlation function was then fit to a single exponential function to obtain the autocorrelation time of the directional persistence. Cells were classified as amoeboid provided the average velocity $<v>\leq 8\;\mathrm{\mu m}\;s^{-1}$ based off the speed distributions (Fig. 2A) and an eccentricity ϵ≥0.2.

### SEM and EDX spectroscopy

Liquid cultures (200 μl) of haploid and amoeboid cells were fixed with 1% (w/v) glutaraldehyde and deposited on hydrophilized 0.01% poly-l-lysine–coated silicon wafers. After 10 min of incubation, the wafers were sequentially immersed for 5 min each in 2.5% glutaraldehyde solution (SOW, 27 practical salinity units), 1% osmium tetroxide, and SOW. Wafers were then passed through an ethanol drying series by sequential immersion in 0, 30, 50, 70, 90, and 100% ethanol for 2 min at each stage and, lastly, three times in water-free ethanol. Wafers were dried using a critical point dryer with a cell monolayer protocol (CPD 931 Tousimis, ETH Zürich Microscope Facility ScopeM) and were fixed to aluminum SEM stubs using silver paint. Samples were degassed for 24 hours and then sputter coated with 4-nm platinum-palladium (CCU-010 Metal Sputter Coater Safematic, ETH Zürich Microscope Facility ScopeM).

SEM imaging and EDX spectroscopy were performed using an extreme-high-resolution TFS Magellan 400 microscope (ETH Zürich Microscope Facility ScopeM) with 50-pA current and an accelerating voltage of 5 kV for imaging and 15 kV for EDX. Scattered electrons were collected and imaged with both an immersion concentric backscatter (CBS) electron detector and secondary through-the-lens (TLD) electron detector. EDX was performed on the amoeboid cells and heavily calcified *C. braarudii*, rather than diploid *G. huxleyi* due to the gradual decalcification of laboratory-maintained *G. huxleyi* cultures (*11*).

### Cell surface characterization

*G. huxleyi* cells of RCC1217 haploid were grown to stationary phase, and amoeboid metamorphosis was induced as described previously (see the “Bacterial growth, amoeboid induction conditions, and exudate preparation” section).

*G. huxleyi* microbial adhesion to hydrocarbons assay (MATH) was performed on the basis of a previously described protocol (*67*). Amoeboid and haploid cultures were filtered with a 1-μm cell filter (pluriStrainer, pluriSelect), with washing and resuspension in phosphate urea magnesium sulfate buffer (*67*) with cell concentrations adjusted to 30 × 10^3^ algae cells/ml. *n*-Hexadecane (150 μl) was then added to 5-ml glass vials containing 1.5 ml of cell suspension and vortexed for 2 min. These samples were allowed to settle for 15 min, and, then, the number of cells in the aqueous hexadecane free layer was measured using the flow cytometry protocol with allophycocyanin A (APC-A; see the “Flow cytometry and staining” section). These cell counts were then compared to cell counts from control blanks without hexadecane for both cultures. The percentage hydrophobicity was then calculated as follows: Hydrophobicity (%) = (*A*_0_ − *A*_1_)/*A*_0_ × 100, where *A*_0,1_ are the algal cell counts in the aqueous layers of hexadecane-free blank and hexadecane-treated samples, respectively.

The zeta potential of *G. huxleyi* amoeboid and haploid samples was measured using a Zetasizer Nano (Malvern). Amoeboid and haploid cultures were filtered with a 1-μm cell filter (pluriStrainer, pluriSelect), with washing and resuspension in fresh L1 medium at a concentration of 3 × 10^4^ cells/ml. Cell suspension (700 μl) was then transferred to a folded capillary zeta cell (DTS1070, Malvern). Zeta potential measurements were performed with automatic voltage selection, monomodal analysis, and a 60-s delay between measurements (*68*).

### PAM microscopy

An amoeboid culture was prepared by inoculating a stationary phase *G. huxleyi* haploid culture with a washed and resuspended *Marinomonas* 5a1 culture (see the “Bacterial growth, amoeboid induction conditions, and exudate preparation” section) and incubated at 18°C on a 14:10 light:dark cycle at 100 μmol m^−2^ s^−1^ for 24 hours before imaging. A 30-μl sample of cell suspension was confined within a silicone gasket (Grace Bio Labs, 0.5-mm thickness) separating two coverslips (Epredia, Number 1.5) coated with 0.1% (w/v) poly-l-lysine solution (Sigma-Aldrich, CAS 25988-63-00) to immobilize the cells. The cells were then dark adapted for 15 min before being loaded onto a chlorophyll-fluorescence imaging microscope (*69*) (Florescence Kinetic Microscope; Photon Systems Instruments), with bright-field and fluorescence images captured with a TOMI 2 camera (Photo Systems Instruments). Bright-field images were used to identify cell positions for extraction of fluorescence parameters from the chlorophyll fluorescence images using a custom python script. The distributions of the maximum and effective quantum yields showed a single population peak, confirming that most of the cells were of one type and not that two separate populations in the amoeboid treatment averaged to the same value (fig. S6). The maximum quantum yield of photosynthetic energy conversion $\frac{F_{v}}{F_{m}}=\frac{F_{m}-F_{0}}{F_{m}}$ and the effective quantum yield of photosynthetic energy conversion $\phi_{\|}=\frac{\left(F_{m}^{\prime }-F\right)}{{F_{m}}^{\prime }}$ were measured using the saturation pulses method (*41*, *70*), where $F_{v},F_{m},\text{and}\;F_{0}$ are the variable, maximal, and minimal fluorescence of dark adapted cells, respectively; F is the fluorescence emission of dark-adapted cells; and ${F_{m}}^{\prime }$ is the maximal fluorescence of light-adapted cells. Rapid light curves were constructed by measuring effective quantum yields at increasing levels of photosynthetically active radiation (PAR). The maximum quantum yield of photosynthetic energy conversion in photosystem II (PSII) provides a relative measure of the maximal efficiency at which light drives PSII photochemistry. The rETR driven by PSII was calculated as $\text{rETR}=\text{PAR}\times \phi_{\|}$ , which estimates the relative rate of realized linear electron flow through PSII at a given PAR intensity.

### Flow cytometry and staining

Algal cells were counted using a flow cytometer (Beckman Coulter, CytoFLEX S) equipped with a 488-nm laser, and FSC and red (APC-A) fluorescence was recorded for each sample for a standardized time per sample of 40 s. Bacterial and algae cultures for determining ploidy were stained and incubated with SYBR Green without any prior cell fixation or nuclei isolation (5 μM final concentration; Sigma-Aldrich, CAS 163795-75-3) for 10 min in the dark and counted using a flow cytometer recording FSC and green (fluorescein isothiocyanate) fluorescence for a standardized time of 60 s per sample. Amoeboid cultures were first filtered with a 1-μm cell filter (pluriStrainer, pluriSelect) and resuspended in filtered medium before counting to prevent machine clogging.

### Cell acidic compartment staining

*G. huxleyi* cells (RCC1217 haploid and CCMP3266 diploid) were grown to stationary phase, and amoeboid metamorphosis was induced overnight in RCC1217 as described above (see the “Bacterial growth, amoeboid induction conditions, and exudate preparation” section). Samples (1 ml) were collected from haploid, diploid, and amoeboid phases and stained with LysoTracker Deep Green DND-26 (Thermo Fisher Scientific, L7526) to a final concentration of 50 nM (*71*). After staining, cells were imaged immediately using a fluorescent microscope (Nikon Ti-E; 30× NA objective, Sola Light Engine Lumencor) and a FITC filter cube (Chroma 49002).

### Statistics and reproducibility

All statistical analyses were done using GraphPad Prism 10.4.1 (GraphPad Software, La Jolla, CA, USA). Data are all reported as the means ± SEM. In all cases, a *P* value of <0.05 was considered statistically significant.

Figure 3A: Statistical significance of the data with *n* = 6 biological replicates was tested using an unpaired two-tailed *t* test with equal variances assumed (*t*_16_ = 0.1501, *P* = 0.8826). Figure 3B: Statistical significance of the data with *n* = 6 biological replicates was tested using an unpaired two-tailed *t* test with equal variances assumed (*t*_10_ = 0.3404, *P* = 0.7406). Figure 3C: To assess whether there was a significant difference between the treatment groups (with *n* = 3 within each treatment), a one-way analysis of variance (ANOVA) was performed (*F*_10,21_ = 111.4, *P* < 0.0001) followed by a one-tailed post hoc Tukey test to compare pairwise group differences against the control treatment (SOW) with a significant difference seen between control group SOW and 5a1 with *P* < 0.0001. Figure 3D: To assess whether there was a significant difference between the treatment groups (with *n* = 3 biological replicates within each treatment), a one-way ANOVA was performed (*F*_14,30_ = 19.67, *P* < 0.0001) followed by a one-tailed post hoc Tukey test to compare pairwise group differences against the control treatment (SOW) with a significant difference seen with 5a1 (*P* < 0.0001), 3g5 (*P* < 0.0001), 3i3 (*P* < 0.0001), and 5a1 (*P* = 0.0234).

Figure 4A: To assess whether there was a significant difference between the treatment groups (with *n* = 3 biological replicates within each treatment), a one-way ANOVA was performed (*F*_4,10_ = 38.97, *P* < 0.0001) followed by a one-tailed post hoc Tukey test to compare pairwise group differences against the control treatment (SOW) with a significant difference seen with 5a1_cells (*P* < 0.0001) and 5a1_ex_SM (*P* = 0.00135). Figure 4B: To assess whether there was a significant difference between the treatment groups (with *n* = 3 biological replicates within each treatment), a one-way ANOVA was performed (*F*_5,12_ = 16.85, *P* ≤ 0.0001) followed by a one-tailed post hoc Tukey test to compare pairwise group differences against the control treatment (SOW) with a significant difference seen with 5a1 + L1 (*P* < 0.00315) and 5a1 + L1 (24 hours) (*P* < 0.0001). Figure 4C: To assess whether there was a significant difference between the treatment groups (with *n* = 3 within each treatment), a one-way ANOVA was performed (*F*_4,10_ = 13.06, *P* = 0.0003) followed by a one-tailed post hoc Tukey test to compare pairwise group differences against the control treatment (SOW) with a significant difference seen in the day 14 group for SOW versus 1 × 10^6^ bacteria cells/ml added (*P* = 0.0002), 10 × 10^6^ bacteria cells/ml added (*P* = 0.0004), and 100 × 10^6^ bacteria cells/ml added (*P* = 0.01255). In the day 20 group, there was a significant difference observed for SOW versus 100 × 10^6^ bacteria cells/ml added (*P* = 0.0011) and 1000 × 10^6^ bacteria cells/ml added (*P* = 0.0032). Figure 4D: To assess whether there was a significant difference between the treatment groups (with *n* = 3 within each treatment), a one-way ANOVA was performed (*F*_4,30_ = 39.47, *P* < 0.0001) followed by a one-tailed post hoc Tukey test to compare pairwise group differences against the control treatment (SOW) with a significant difference seen in the no dilution group for SOW versus 100 × 10^6^ bacteria cells/ml added (*P* < 0.0001) and 1000 × 10^6^ bacteria cells/ml added (*P* < 0.0001). In the 5× dilution group, there was a significant difference observed for SOW versus 100 × 10^6^ bacteria cells/ml added (*P* = 0.0657) and 1000 × 10^6^ bacteria cells/ml added (*P* < 0.0001). In the 10× dilution group, there was a significant difference observed for SOW versus 10 × 10^6^ bacteria cells/ml added (*P* = 0.0068) and 100 × 10^6^ bacteria cells/ml added (*P* < 0.0001).

Figure S2A: Statistical significance of the data with *n* = 3 biological replicates was tested using an unpaired two-tailed *t* test with equal variances assumed (*t*_4_ = 0.9748, *P* = 0.3849). Figure S2B: Statistical significance of the data with *n* = 5 biological replicates was tested using an unpaired two-tailed *t* test with equal variances assumed (*t*_8_ = 16.43, *P* < 0.001).

## Supplementary Materials

### The PDF file includes:

Figs. S1 to S7 Legends for movies S1 and S2 References
