# Supplementary material for: Bacteria induce an amoeboid phase in coccolithophores that persists after bloom collapse
Source: Sci Adv. 2025 Aug 27;11(35):eadw7280. doi: 10.1126/sciadv.adw7280 (PMC12383271; doi:10.1126/sciadv.adw7280)
Supplement: Supplementary file 1 — Figs. S1 to S7 Legends for movies S1 and S2 References [file sciadv.adw7280_sm.pdf]

Science Advances
AAAS

Supplementary Materials for

Bacteria induce an amoeboid phase in coccolithophores that persists after bloom collapse

Sophie T. Zweifel *et al.*

Corresponding author: Roman Stocker, romanstocker@ethz.ch

Sci. Adv. **11**, eadw7280 (2025)
DOI: 10.1126/sciadv.adw7280

The PDF file includes:

Figs. S1 to S7
Legends for movies S1 and S2
References

Other Supplementary Material for this manuscript includes the following:

Movies S1 and S2

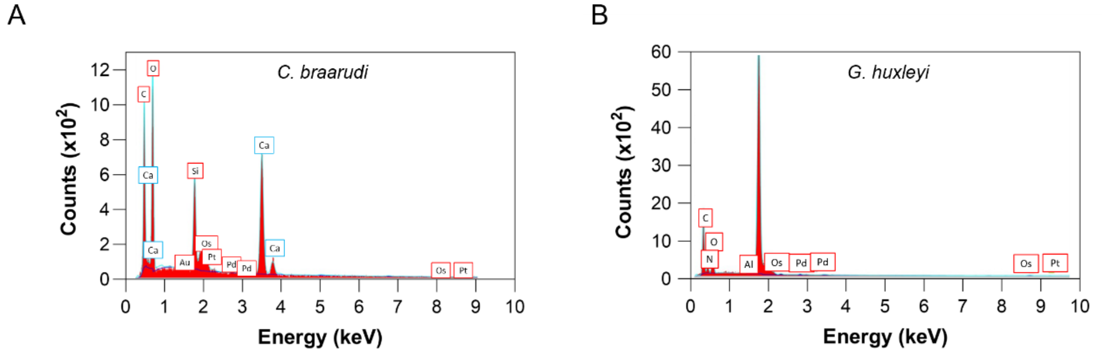

Supplementary Figure S1. **Energy-dispersive X-ray (EDX) spectra of diploid *Coccolithus braarudii* and amoeboid *Geopharycapsa huxleyi*.** (A) Characteristic peaks of calcium carbonate, indicating cell calcification in *C. braarudii* but not in (B) *G. huxleyi*.

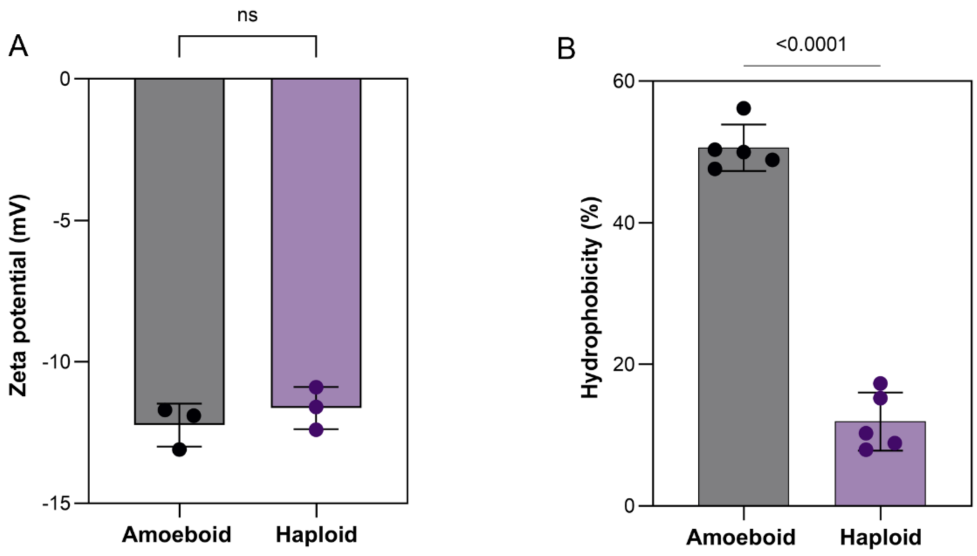

Supplementary Figure S2. **Comparison of surface properties between *G. huxleyi* haploid and amoeboid phases.** (A) Zeta potential was measured for the haploid and amoeboid morphotype. Data shown as the mean with n = 3 biological replicates with SE error bars. A 2-tailed t-test was applied for the hydrocarbon-free treatment against the blank hydrocarbon-free treatment ($p < 0.05$). (B) A microbial adhesion to hydrocarbon (MATH) assay was performed on haploid and amoeboid *G. huxleyi* revealing an increase of 39 ± 2% hydrophobicity in the amoeboid phase. A 2-tailed t-test was applied for the hydrocarbon-free treatment against the blank hydrocarbon-free treatment ($p < 0.05$). Data shown as the mean with n = 5 biological replicates with SE error bars.

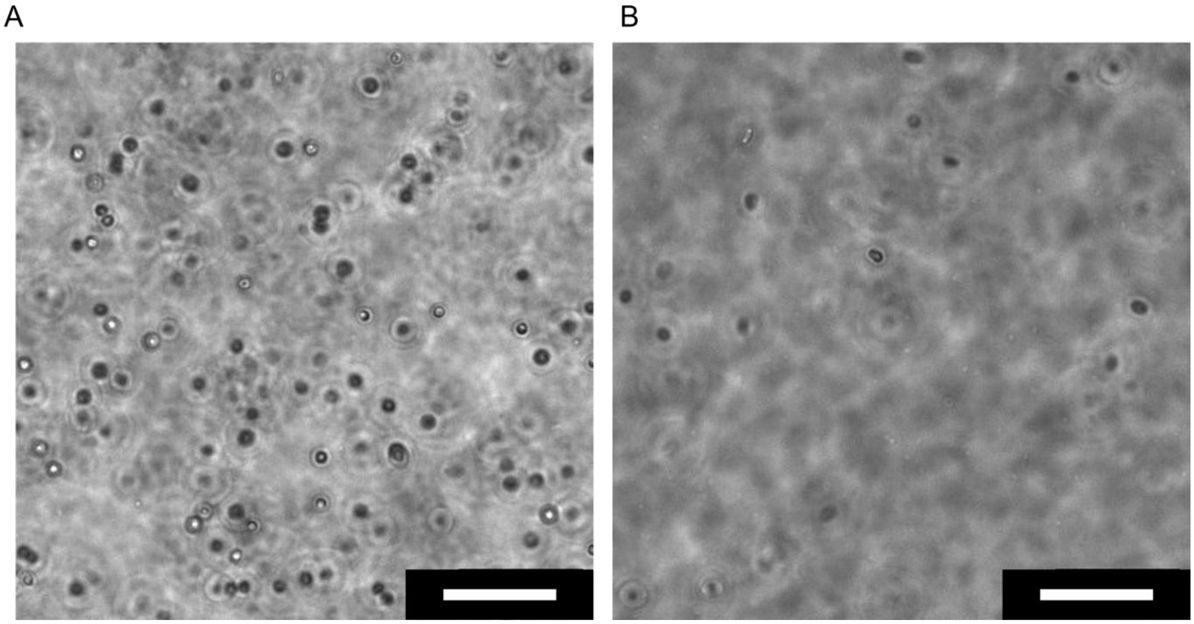

Supplementary Figure S3. **Light microscopy images of (*A*) haploid and (*B*) amoeboid *Geopharycapsa oceanica.*** (A) In control conditions haploid *G. oceanica* cells show a spherical morphology. (B) In treatment conditions where haploid *G. oceanica* cultures were exposed to *G. huxleyi*-associated bacteria, the algal cells underwent a morphological transformation from spherical to an elongated cell morphology. Scale bars represent 50 µm**.**

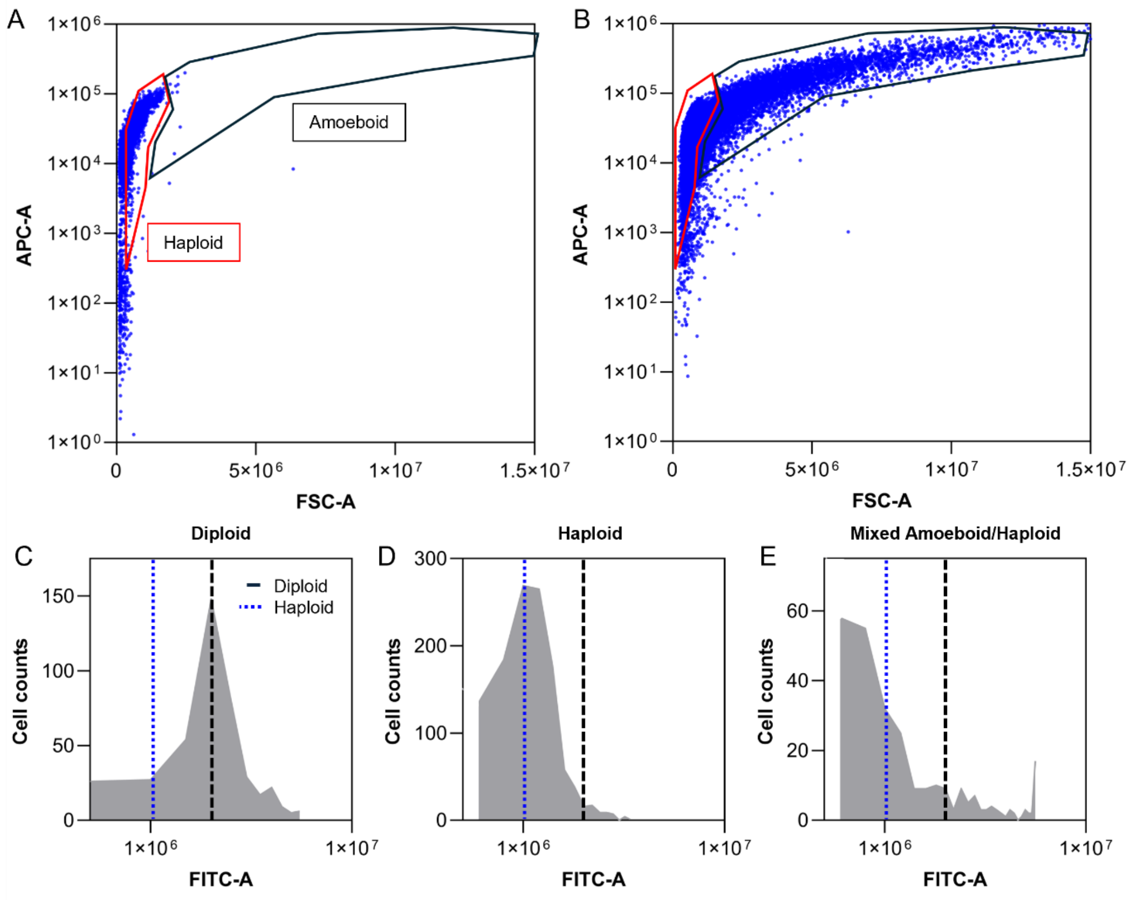

Supplementary Figure S4. **Flow cytometry gating for the identification of *G. huxleyi* amoeboid cells and FITC-A distributions for ploidy determination.** Autofluorescence chlorophyll APC-A fluorescent signal for *G. huxleyi* (A) haploid and (B) mixed haploid and amoeboid cultures. Ploidy was determined from the FITC-A fluorescence distribution signal obtained from SYBR green staining of *G. huxleyi* cultures, identifying diploid/haploid characteristic FITC-A intensities (black dashed line and dotted blue lines respectively) for the following *G. huxleyi* cultures: (C) diploid, (D) haploid, and (E) mixed amoeboid and haploid.

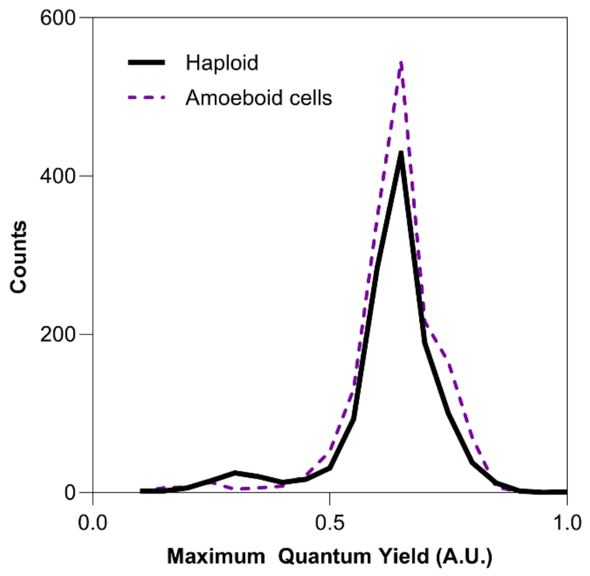

Supplementary Figure S5. **Maximum quantum yield distribution curves for control and bacteria-treated *G. huxleyi* cultures**. Maximum quantum yields were obtained using Pulse Amplitude Modulation (PAM) microscopy with analysis areas selected using light microscopy to determine amoeboid-enriched areas. The absence of a substantial second peak in either of the conditions confirms that there was a single dominant population of cells.

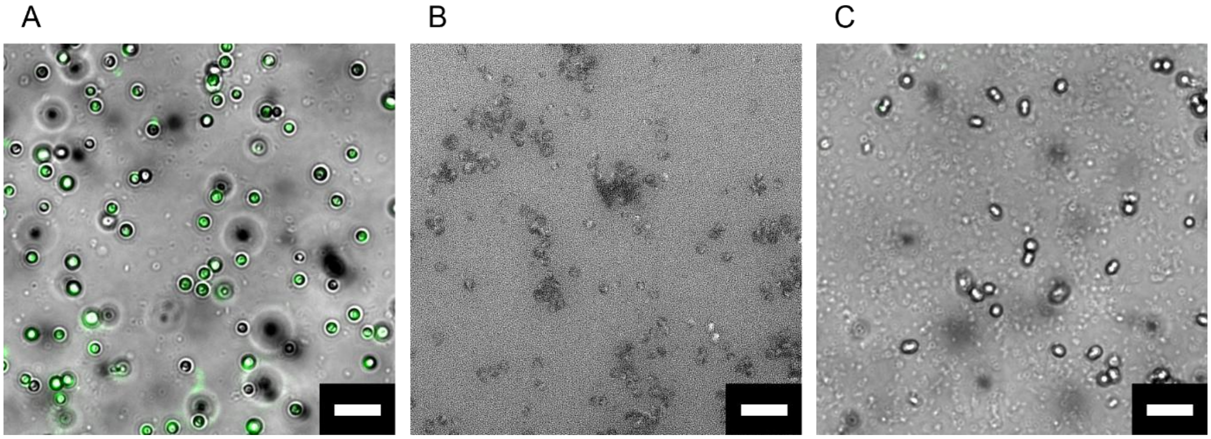

Supplementary Figure S6. **Fluorescence microscopy images of *G. huxleyi* cells stained for the acid compartment using LysoTracker™ Deep Green DND 26.** Staining of the acidic compartment reveals signal in the haploid phase (*A*) but not in the diploid *(B)* or the amoeboid *(C)* phases, consistent with reports of phagocytosis by haploids (*46*). The lack of staining in diploid *G. huxleyi* confirmed that the acidic compartments involved in calcification were not stained through this method and therefore the staining observed in the haploid (*A*) was not due to these acidic compartments (*72, 73*). Scale bars represent 20 µm.

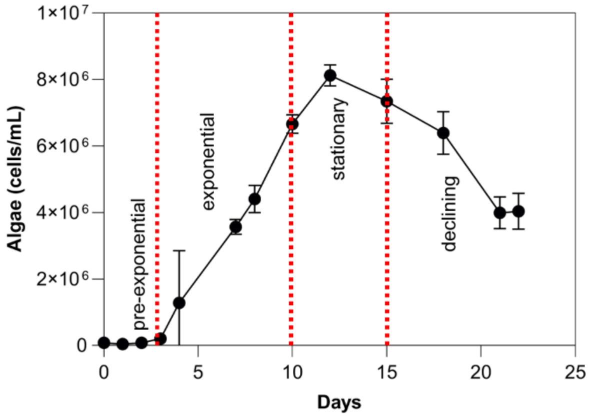

Supplementary Figure S7. **Growth curve of *Geopharycapsa huxleyi* RCC1217.** Cell counts of haploid *G. huxleyi* cultures were measured using flow cytometry (see Methods) to identify the growth phases. Data shown as the mean with SE error bars and n = 3 biological replicates.

Supplementary Video S1. **Transformation of haploid *Geopharycapsa huxleyi* into the amoeboid morphotype.** Timelapse microscopy video (two frames per min) showing transformation of a spherical haploid *G. huxleyi* cell into an elongated "amoeboid" cell type over a period of approximately 16 min.

Supplementary Video S2. **Swimming of amoeboid *Geopharycapsa huxleyi.*** Light microscopy video (20 fps) of *G. huxleyi* culture treated with bacteria 24 h prior to imaging, illustrating the clear cell body rotation of the amoeboid cell.

REFERENCES AND NOTES

1. C. B. Field, M. J. Behrenfeld, J. T. Randerson, P. Falkowski, Primary production of the biosphere: Integrating terrestrial and oceanic components. *Science* **281**, 237–240 (1998).

2. M. C. Carvalho, K. G. Schulz, B. D. Eyre, Respiration of new and old carbon in the surface ocean: Implications for estimates of global oceanic gross primary productivity. *Global Biogeochem. Cycles* **31**, 975–984 (2017).

3. A. J. Poulton, T. R. Adey, W. M. Balch, P. M. Holligan, Relating coccolithophore calcification rates to phytoplankton community dynamics: Regional differences and implications for carbon export. *Deep Sea Res. Part II Top. Stud. Oceanogr.* **54**, 538–557 (2007).

4. A. J. Poulton, S. C. Painter, J. R. Young, N. R. Bates, B. Bowler, D. Drapeau, E. Lyczsckowski, W. M. Balch, The 2008 *Emiliania huxleyi* bloom along the Patagonian Shelf: Ecology, biogeochemistry, and cellular calcification. *Global Biogeochem. Cycles* **27**, 1023–1033 (2013).

5. J. D. Milliman, Production and accumulation of calcium carbonate in the ocean: Budget of a nonsteady state. *Global Biogeochem. Cycles* **7**, 927–957 (1993).

6. K. M. Krumhardt, N. S. Lovenduski, M. C. Long, M. Levy, K. Lindsay, J. K. Moore, C. Nissen, Coccolithophore growth and calcification in an acidified ocean: Insights from community earth system model simulations. *J. Adv. Model Earth Syst.* **11**, 1418–1437 (2019).

7. W. M. Balch, The ecology, biogeochemistry, and optical properties of coccolithophores. *Ann. Rev. Mar. Sci.* **10**, 71–98 (2018).

8. S. A. O'Dea, S. J. Gibbs, P. R. Bown, J. R. Young, A. J. Poulton, C. Newsam, P. A. Wilson, Coccolithophore calcification response to past ocean acidification and climate change. *Nat. Commun.* **5**, 5363 (2014).

9. E. M. Bendif, B. Nevado, E. L. Y. Wong, K. Hagino, I. Probert, J. R. Young, R. E. M. Rickaby, D. A. Filatov, Repeated species radiations in the recent evolution of the key marine phytoplankton lineage *Gephyrocapsa*. *Nat. Commun.* **10**, 4234 (2019).

10. H. C. Wong, P. Wang, Induction of viable but nonculturable state in *Vibrio parahaemolyticus* and its susceptibility to environmental stresses. *J. Appl. Microbiol.* **96**, 359–366 (2004).

11. E. Segev, T. P. Wyche, K. H. Kim, J. Petersen, C. Ellebrandt, H. Vlamakis, N. Barteneva, J. N. Paulson, L. Chai, J. Clardy, R. Kolter, Dynamic metabolic exchange governs a marine algal-bacterial interaction. *eLife* **5**, e17473 (2016).

12. C. Martínez-Pérez, S. T. Zweifel, R. Pioli, R. Stocker. Space, the final frontier: The spatial component of phytoplankton-bacterial interactions. *Mol. Microbiol.* **122**, 331–346 (2024).

13. D. Schatz, S. Rosenwasser, S. Malitsky, S. G. Wolf, E. Feldmesser, A. Vardi, Communication via extracellular vesicles enhances viral infection of a cosmopolitan alga. *Nat. Microbiol.* **2**, 1485–1492 (2017).

14. D. Schatz, G. Schleyer, M. R. Saltvedt, R.-A. Sandaa, E. Feldmesser, A. Vardi, Ecological significance of extracellular vesicles in modulating host-virus interactions during algal blooms. *ISME J.* **15**, 3714–3721 (2021).

15. D. G. Mann, The species concept in diatoms. *Phycologia* **38**, 437–495 (1999).

16. D. Klaveness, *Coccolithus huxleyi* (Lohm.) Kamptn II. The flagellate cell, aberrant cell types, vegetative propagation and life cycles. *Br. Phycol. J.* **7**, 309–318 (1972).

17. J. C. Green, P. A. Course, G. A. Tarran, The life-cycle of *Emiliania huxleyi*: A brief review and a study of relative ploidy levels analysed by flow cytometry. *J. Mar. Syst.* **9**, 33–44 (1996).

18. M. Parke, I. Adams, The motile (*Crystallolithus hyalinus* Gaarder & Markali) and non-motile phases in the life history of *Coccolithus pelagicus* (Wallich) Schiller. *J. Mar. Biol. Assoc. U.K.* **39**, 263–274 (1960).

19. A. Houdan, C. Billard, D. Marie, F. Not, A. G. Sáez, J. R. Young, I. Probert, Holococcolithophore-heterococcolithophore (Haptophyta) life cycles: Flow cytometric analysis of relative ploidy levels. *Syst. Biodivers.* **1**, 453–465 (2004).

20. W. Eikrem, L. K. Medlin, J. Henderiks, S. Rokitta, B. Rost, I. Probert, J. Throndsen, B. Edvardsen, "Haptophyta," in *Handbook of the Protists*, J. M. Archibald, A. G. B. Simpson, C. H. Slamovits, L. Margulis, M. Melkonian, D. J. Chapman, J. O. Corliss, Eds. (Springer International Publishing, 2017), pp. 1–61.

21. J. de Vries, F. Monteiro, G. Wheeler, A. Poulton, J. Godrijan, F. Cerino, E. Malinverno, G. Langer, C. Brownlee, Haplo-diplontic life cycle expands coccolithophore niche. *Biogeosciences* **18**, 1161–1184 (2021).

22. D. A. Filatov, E. M. Bendif, O. A. Archontikis, K. Hagino, R. E. M. Rickaby, The mode of speciation during a recent radiation in open-ocean phytoplankton. *Curr. Biol.* **31**, 5439–5449.e5 (2021).

23. M. J. Frada, K. D. Bidle, I. Probert, C. de Vargas, In situ survey of life cycle phases of the coccolithophore *Emiliania huxleyi* (*Haptophyta*). *Environ. Microbiol.* **14**, 1558–1569 (2012).

24. T. Tyrrell, A. Merico, "*Emiliania huxleyi*: Bloom observations and the conditions that induce them," in *Coccolithophores: From Molecular Processes to Global Impact*, H. R. Thierstein, J. R. Young, Eds. (Springer Berlin Heidelberg, 2004), pp. 75–97.

25. J.-P. Gattuso, R. W. Buddemeier, Ocean biogeochemistry. Calcification and CO_2. *Nature* **407**, 311–313 (2000).

26. P. M. Holligan, M. Viollier, D. S. Harbour, P. Camus, M. Champagne-Philippe, Satellite and ship studies of coccolithophore production along a continental shelf edge. *Nature* **304**, 339–342 (1983).

27. P. M. Holligan, E. Fernández, J. Aiken, W. M. Balch, P. Boyd, P. H. Burkill, M. Finch, S. B. Groom, G. Malin, K. Muller, D. A. Purdie, C. Robinson, C. C. Trees, S. M. Turner, P. van der Wal, A biogeochemical study of the coccolithophore, *Emiliania huxleyi*, in the North Atlantic. *Global Biogeochem. Cycles* **7**, 879–900 (1993).

28. D. E. Raitsos, S. J. Lavender, Y. Pradhan, T. Tyrrell, P. C. Reid, M. Edwards, Coccolithophore bloom size variation in response to the regional environment of the subarctic North Atlantic. *Limnol. Oceanogr.* **51**, 2122–2130 (2006).

29. M. D. Iglesias-Rodriguez, R. Armstrong, R. Feely, R. Hood, J. Kleypas, J. D. Milliman, C. Sabine, J. Sarmiento, Progress made in study of ocean's calcium carbonate budget. *Eos Trans. AGU* **83**, 365–375 (2002).

30. W. H. Wilson, G. A. Tarran, D. Schroeder, M. Cox, J. Oke, G. Malin, Isolation of viruses responsible for the demise of an *Emiliania huxleyi* bloom in the English Channel. *J. Mar. Biol. Assoc. U.K.* **82**, 369–377 (2002).

31. G. Bratbak, J. K. Egge, M. Heldal, Viral mortality of the marine alga *Emiliania huxleyi* (Haptophyceae) and termination of algal blooms. *Mar. Ecol. Prog. Ser.* **93**, 39–48 (1993).

32. G. Bratbak, W. Wilson, M. Heldal, Viral control of *Emiliania huxleyi* blooms? *J. Mar. Syst.* **9**, 75–81 (1996).

33. M. Trainic, I. Koren, S. Sharoni, M. Frada, L. Segev, Y. Rudich, A. Vardi, Infection dynamics of a bloom-forming alga and its virus determine airborne coccolith emission from seawater. *iScience* **6**, 327–335 (2018).

34. M. Frada, I. Probert, M. J. Allen, W. H. Wilson, C. de Vargas, The "Cheshire Cat" escape strategy of the coccolithophore *Emiliania huxleyi* in response to viral infection. *Proc. Natl. Acad. Sci. U.S.A.* **105**, 15944–15949 (2008).

35. G. J. Mordecai, F. Verret, A. Highfield, D. C. Schroeder, Schrödinger's Cheshire Cat: Are haploid *Emiliania huxleyi* cells resistant to viral infection or not? *Viruses* **9**, 51 (2017).

36. M. J. Frada, S. Rosenwasser, S. Ben-Dor, A. Shemi, H. Sabanay, A. Vardi, Morphological switch to a resistant subpopulation in response to viral infection in the bloom-forming coccolithophore *Emiliania huxleyi*. *PLOS Pathog.* **13**, e1006775 (2017).

37. T. Braarud, Reproduction in the marine coccolithophorid Coccolithus huxleyi in culture. *Pubbl. Stn. Zool. Napoli* **33**, 110–116 (1963).

38. D. H. Green, V. Echavarri-Bravo, D. Brennan, M. C. Hart, Bacterial diversity associated with the coccolithophorid algae *Emiliania huxleyi* and *Coccolithus pelagicus* f. *braarudii*. *Biomed. Res. Int.* **2015**, e194540 (2015).

39. T. Koga, N. Okahashi, I. Takahashi, T. Kanamoto, H. Asakawa, M. Iwaki, Surface hydrophobicity, adherence, and aggregation of cell surface protein antigen mutants of Streptococcus mutans serotype c. *Infect. Immun.* **58**, 289 (1990).

40. R. Vij, C. Danchik, C. Crawford, Q. Dragotakes, A. Casadevall, Variation in cell surface hydrophobicity among *Cryptococcus neoformans* strains influences interactions with amoebas. *mSphere* **5**, e00310 (2020).

41. N. R. Baker, Chlorophyll fluorescence: A probe of photosynthesis in vivo. *Annu. Rev. Plant Biol.* **59**, 89–113 (2008).

42. S. H. Oh, Y. K. Chang, J. H. Lee, Identification of significant proxy variable for the physiological status affecting salt stress-induced lipid accumulation in Chlorella sorokiniana HS1. *Biotechnol. Biofuels* **12**, 242 (2019).

43. E. H. Murchie, T. Lawson, Chlorophyll fluorescence analysis: A guide to good practice and understanding some new applications. *J. Exp. Bot.* **64**, 3983–3998 (2013).

44. R. P. Barbagallo, K. Oxborough, K. E. Pallett, N. R. Baker, Rapid, noninvasive screening for perturbations of metabolism and plant growth using chlorophyll fluorescence imaging. *Plant Physiol.* **132**, 485–493 (2003).

45. E. E. Clerc, J.-B. Raina, J. M. Keegstra, Z. Landry, S. Pontrelli, U. Alcolombri, B. S. Lambert, V. Anelli, F. Vincent, M. Masdeu-Navarro, A. Sichert, F. De Schaetzen, U. Sauer, R. Simó, J.-H. Hehemann, A. Vardi, J. R. Seymour, R. Stocker, Strong chemotaxis by marine bacteria towards polysaccharides is enhanced by the abundant organosulfur compound DMSP. *Nat. Commun.* **14**, 8080 (2023).

46. Y. Avrahami, M. J. Frada, Detection of phagotrophy in the marine phytoplankton group of the coccolithophores (Calcihaptophycidae, Haptophyta) during nutrient-replete and phosphate-limited growth. *J. Phycol.* **56**, 1103–1108 (2020).

47. A. R. Bramucci, L. Labeeuw, F. D. Orata, E. M. Ryan, R. R. Malmstrom, R. J. Case. The bacterial symbiont *Phaeobacter inhibens* shapes the life history of its algal host *Emiliania huxleyi*. *Front. Mar. Sci.* **5**, 188 (2018).

48. L. Labeeuw, J. Khey, A. R. Bramucci, H. Atwal, A. P. de la Mata, J. Harynuk, R. J. Case, Indole-3-acetic acid is produced by *Emiliania huxleyi* coccolith-bearing cells and triggers a physiological response in bald cells. *Front. Microbiol.* **7**, 828 (2016).

49. J. Zhou, M. L. Richlen, T. R. Sehein, D. M. Kulis, D. M. Anderson, Z. Cai, Microbial community structure and associations during a marine dinoflagellate bloom. *Front. Microbiol.* **9**, 1201 (2018).

50. C. P. D. Brussaard, R. S. Kempers, A. J. Kop, R. Riegman, M. Heldal, Virus-like particles in a summer bloom of *Emiliania huxleyi* in the North Sea. *Aquat. Microb. Ecol.* **10**, 105–113 (1996).

51. N. Barak-Gavish, M. J. Frada, C. Ku, P. A. Lee, G. R. DiTullio, S. Malitsky, A. Aharoni, S. J. Green, R. Rotkopf, E. Kartvelishvily, U. Sheyn, D. Schatz, A. Vardi, Bacterial virulence against an oceanic bloom-forming phytoplankter is mediated by algal DMSP. *Sci. Adv.* **4**, eaau5716 (2018).

52. A. Calbet, M. R. Landry, Phytoplankton growth, microzooplankton grazing, and carbon cycling in marine systems. *Limnol. Oceanogr.* **49**, 51–57 (2004).

53. X. Mayali, F. Azam, Algicidal bacteria in the sea and their impact on algal blooms. *J. Eukaryot. Microbiol.* **51**, 139–144 (2004).

54. N. Novosel, T. Mišić Radić, J. Zemla, M. Lekka, A. Čačković, D. Kasum, T. Legović, P. Žutinić, M. Gligora Udovič, N. Ivošević DeNardis, Temperature-induced response in algal cell surface properties and behaviour: An experimental approach. *J. Appl. Phycol.* **34**, 243–259 (2022).

55. L. O. Villacorte, Y. Ekowati, T. R. Neu, J. M. Kleijn, H. Winters, G. Amy, J. C. Schippers, M. D. Kennedy, Characterisation of algal organic matter produced by bloom-forming marine and freshwater algae. *Water Res.* **73**, 216–230 (2015).

56. K. Xu, Y. Li, X. Zou, H. Wen, Z. Shen, X. Ren, Investigating microalgae cell-microsphere interactions during microalgae harvesting by ballasted dissolved air flotation through XDLVO theory. *Biochem. Eng. J.* **137**, 294–304 (2018).

57. E. Paasche, A review of the coccolithophorid *Emiliania huxleyi* (Prymnesiophyceae), with particular reference to growth, coccolith formation, and calcification-photosynthesis interactions. *Phycologia* **40**, 503–529 (2001).

58. F. Vincent, M. Gralka, G. Schleyer, D. Schatz, M. Cabrera-Brufau, C. Kuhlisch, A. Sichert, S. Vidal-Melgosa, K. Mayers, N. Barak-Gavish, J. M. Flores, M. Masdeu-Navarro, J. K. Egge, A. Larsen, J.-H. Hehemann, C. Marrasé, R. Simó, O. X. Cordero, A. Vardi, Viral infection switches the balance between bacterial and eukaryotic recyclers of organic matter during coccolithophore blooms. *Nat. Commun.* **14**, 510 (2023).

59. S. W. Wilhelm, C. A. Suttle, Viruses and nutrient cycles in the sea: Viruses play critical roles in the structure and function of aquatic food webs. *Bioscience* **49**, 781–788 (1999).

60. R. J. Olson, H. M. Sosik, A submersible imaging-in-flow instrument to analyze nano-and microplankton: Imaging FlowCytobot. *Limnol. Oceanogr. Methods* **5**, 195–203 (2007).

61. J. E. Bissinger, D. J. S. Montagnes, J. Harples, D. Atkinson, Predicting marine phytoplankton maximum growth rates from temperature: Improving on the Eppley curve using quantile regression. *Limnol. Oceanogr.* **53**, 487–493 (2008).

62. R. W. Eppley, Temperature and phytoplankton growth in the sea. *Fisch. Bull.* 70 (1972).

63. W. Sunda, N. Price, F. Morel, F. Morel, *Algal Culturing Techniques* (Academic Press, 2005).

64. D. C. Schroeder, J. Oke, G. Malin, W. H. Wilson, Coccolithovirus (*Phycodnaviridae*): Characterisation of a new large dsDNA algal virus that infects *Emiliana huxleyi*. *Arch. Virol.* **147**, 1685–1698 (2002).

65. M. Homola, C. R. Büttner, T. Füzik, P. Křepelka, R. Holbová, J. Nováček, M. L. Chaillet, J. Žák, D. Grybchuk, F. Förster, W. H. Wilson, D. C. Schroeder, P. Plevka, Structure and replication cycle of a virus infecting climate-modulating alga *Emiliania huxleyi*. *Sci. Adv.* **10**, eadk1954 (2024).

66. D. B. Allan, T. Caswell, N. C. Keim, C. M. van der Wel, R. W. Verweij, soft-matter/trackpy: v0.6.1, Zenodo (2023); https://doi.org/10.5281/zenodo.7670439.

67. Vandana, S. Das, Cell surface hydrophobicity and petroleum hydrocarbon degradation by biofilm-forming marine bacterium *Pseudomonas furukawaii* PPS-19 under different physicochemical stressors. *J. Hazard. Mater.* **457**, 131795 (2023).

68. A. J. Wyness, D. M. Paterson, E. C. Defew, M. I. Stutter, L. M. Avery, The role of zeta potential in the adhesion of *E. coli* to suspended intertidal sediments. *Water Res.* **142**, 159–166 (2018).

69. H. Küpper, I. Šetlík, M. Trtílek, L. Nedbal, A microscope for two-dimensional measurements of in vivo chlorophyll fluorescence kinetics using pulsed measuring radiation, continuous actinic radiation, and saturating flashes. *Photosynthetica* **38**, 553–570 (2000).

70. C. Klughammer, U. Schreiber, "Saturation pulse method for assessment of energy conversion in PS I" (PAM Application Notes, Heinz Walz GmbH, 2008).

71. E. Sintes, P. A. del Giorgio, Community heterogeneity and single-cell digestive activity of estuarine heterotrophic nanoflagellates assessed using lysotracker and flow cytometry. *Environ. Microbiol.* **12**, 1913–1925 (2010).

72. A. R. Taylor, C. Brownlee, G. Wheeler, Coccolithophore cell biology: Chalking up progress. *Ann. Rev. Mar. Sci.* **9**, 283–310 (2017).

73. C. Brownlee, G. L. Wheeler, A. R. Taylor, Coccolithophore biomineralization: New questions, new answers. *Semin. Cell Dev. Biol.* **46**, 11–16 (2015).
